# Inpatient EHR User Experience and Hospital EHR Safety Performance

**DOI:** 10.1001/jamanetworkopen.2023.33152

**Published:** 2023-09-11

**Authors:** David C. Classen, Christopher A. Longhurst, Taylor Davis, Julia Adler Milstein, David W. Bates

**Affiliations:** 1 Division of Epidemiology, University of Utah School of Medicine, Salt Lake City; 2 IDEAS Center, VA Salt Lake City Healthcare System, Salt Lake City, Utah; 3 Department of Medicine, UC San Diego Health, San Diego, California; 4 Department of Pediatrics, UC San Diego Health, San Diego, California; 5 KLAS Enterprises, LLC, Orem, Utah; 6 University of California San Francisco Center for Clinical Informatics and Improvement Research, San Francisco; 7 Division of General Internal Medicine and Primary Care, Department of Medicine, Brigham and Women’s Hospital, Boston, Massachusetts; 8 Department of Health Policy and Management, Harvard T.H. Chan School of Public Health, Boston, Massachusetts

## Abstract

**IMPORTANCE:**

Despite the broad adoption and optimization of electronic health record (EHR) systems across the continuum of care, serious usability and safety problems persist.

**OBJECTIVE:**

To assess whether EHR safety performance is associated with EHR frontline user experience in a national sample of hospitals.

**DESIGN, SETTING, AND PARTICIPANTS:**

This cross-sectional study included all US adult hospitals that used the National Quality Forum Leapfrog Health IT Safety Measure and also used the ARCH Collaborative EHR User experience survey from January 1, 2017, to January 1, 2019. Data analysis was performed from September 2020 to November 2022.

**MAIN OUTCOMES AND MEASURES:**

The primary outcomes were hospital performance on the Leapfrog Health IT Safety measure (overall and 10 subcomponents) and the ARCH collaborative frontline user experience scores (overall and 8 subcomponents). Ordinary least squares models with survey responses clustered by hospital were used to assess associations between the overall measures and their subcomponents.

**RESULTS:**

There were 112 hospitals and 5689 frontline user surveys included in the study. Hospitals scored a mean of 0.673 (range, 0.297-0.973) on the Leapfrog Health IT safety measure; the mean ARCH EHR user experience score was 3.377 (range, 1 [best] to 5 [worst]). The adjusted β coefficient between the overall safety score and overall user experience score was 0.011 (95% CI, 0.006-0.016). The ARCH overall score was also significantly associated with 10 subcategory scores of the Leapfrog Health IT safety score, and the overall Leapfrog score was associated with the 8 subcategory scores of the ARCH user experience score.

**CONCLUSIONS AND RELEVANCE:**

This cross-sectional study found a positive association between frontline user–rated EHR usability and EHR safety performance. This finding suggests that improving EHR usability, which is a current well-known pain point for EHR users, could have direct benefits in terms of improved EHR safety.

## Key Points

**Question:**

Is the safety performance of electronic health record (EHR) systems associated with frontline usability of such systems?

**Findings:**

In this cross-sectional study of 112 US hospitals from 2017 and 2018, there was a significant association between the overall scores of the National Quality Forum Health IT Safety Measure, a computerized physician order entry and EHR safety test, and the ARCH Collaborative EHR User Experience Survey. In addition, there was an association between the overall EHR Safety Test Score and the subcategory scores on the ARCH Survey mean scores and the overall ARCH Survey score and the subcomponent scores in the EHR Safety Score.

**Meaning:**

These findings suggest that EHR safety performance is associated with frontline EHR usability and that current broad efforts to improve EHR usability may be associated with improvements in EHR safety performance as well.

## Teaser

This cross-sectional study examines whether electronic health record (EHR) system safety performance is associated with EHR frontline user experience in a national sample of hospitals.

## Introduction

The US health care system has broadly adopted electronic health record (EHR) systems, with over 95% of hospitals and more than 90% of ambulatory clinics having implemented these systems.^1^ Although improvements in patient safety were widely expected, given the demonstrated ability of EHRs to reduce medication errors, commercial EHR systems have largely failed to consistently deliver this benefit.^1,2^ A recent study^1^ using a national sample of hospitals found that, over the last 10 years, EHRs have failed to substantially improve their medication safety performance, with 33% of known serious medications errors still not prevented in 2018. One explanation for these results has been poor EHR system usability, which has been shown to negatively affect the safety of these systems, not only failing to prevent, but in several cases leading to, medication errors.^3^ Clinical decision support usability in EHRs is often poorly designed and difficult to use, resulting in high levels of alerts being overridden in commercial EHRs.^1,2,3^

EHR usability can be defined as the effectiveness, efficiency, and satisfaction with which specified users achieve specified goals in particular environments. Although many studies on EHR usability have been published, few have focused on its relationship with safety. A recent review^4^ found that few usability studies explicitly examined the implications for safety and that most studies did not link usability challenges to specific safety outcomes. One study^5^ compared medication-related alerts among EHRs and showed substantial variation in usability. It has also been demonstrated that the likelihood of acceptance of medication-related decision support is associated with usability factors, such as quality of display of the alerts.^6^ Despite these important findings, there is limited evidence directly linking a source of insight into usability—frontline user experiences with EHRs—and either safety outcomes or safety performance of EHRs. Frontline users are increasingly being asked to report their perceptions of EHR usability, and these reports could help identify challenges and opportunities to improve EHRs.^7,8,9^ Although prior studies have reported these user perceptions of usability, it is not known whether or to what degree user perceptions are associated with EHR safety performance. To the extent they are associated, it is likely that EHRs with poor usability have less-effective medication ordering and less reduction of medication errors.^10,11^ Of note, one recent study^12^ outlined a new EHR safety and usability joint assessment.

In this study, we leveraged a large-scale survey of frontline user perceptions of EHR usability, including responses from physicians, nurses, advanced practice professionals, and residents across almost 300 hospitals.^13^ This initiative, led by the ARCH Collaborative (KLAS Research, Pleasant Grove, Utah), focused solely on inpatient care has been ongoing for more than 5 years, and 1 dimension of the survey focuses on the extent to which the respondent’s EHR supports the overall goal of the delivery of high-quality, safe care. To examine associations with objective EHR safety performance, we leveraged a nationally endorsed EHR safety test, the National Quality Forum Leapfrog computerized provider order entry (CPOE) and EHR safety test. As a unique linkage of these 2 large-scale data sets, our study contributes to the evolving understanding of the association of frontline user perceptions of EHR usability with EHR safety performance. The results may be particularly useful to EHR vendors and health systems that regularly collect frontline user feedback, to help them better understand how to use that information to improve the safety performance of EHR systems.

## Methods

This cross-sectional study follows the Strengthening the Reporting of Observational Studies in Epidemiology (STROBE) reporting guidelines. This study was reviewed by the University of Utah institutional review board and was determined to be exempt from the need for informed consent because no individual patient data were used, in accordance with 45 CFR §46.

### Data

#### Leapfrog EHR Safety Evaluation Test

The Leapfrog EHR/CPOE Safety Evaluation Test was designed by investigators at the University of Utah and the Brigham and Women’s Hospital and is used by the Leapfrog Group as part of their annual, national hospital survey that promotes performance transparency and improvement. It is a free, annual survey distributed nationally to US hospitals, with the results reported publicly every year. The results from the EHR/CPOE Evaluation Safety Test represent just one of several metrics used by the Leapfrog Group in describing the quality and safety of care that hospitals deliver to purchasers, consumers, and users of EHRs.^1^ The EHR/CPOE Evaluation Safety Test is endorsed by the National Quality Forum as part of their Safe Practices for Better Healthcare report,^1^ which includes an EHR/CPOE standard. The Leapfrog EHR/CPOE Evaluation Test is a simulation test that uses simulated test patients and test medication orders to mimic the experience of a physician writing orders for actual patients to evaluate EHR safety performance. The order types are divided into 2 categories: orders with potential adverse events prevented by basic clinical decision support (drug-allergy, drug-route, drug-drug, drug-dose for single doses, and therapeutic duplication) and those that require advanced decision support (drug-laboratory, drug-dose for daily doses, drug-age, drug-diagnosis, and corollary orders). The primary outcome measure is whether the hospital EHR/CPOE system correctly generated an alert after entering a test order that could have caused an adverse drug event. The hospital takes the test and immediately receives an overall test score and 10 subcategory scores that are recorded as part of the overall Leapfrog Hospital Safety Survey. The overall test score is the number of questions on the test that the hospital got correct, expressed as a decimal from 0 to 1. This test has been described in detail elsewhere.^1^

#### EHR Usability Experience Measurement

The KLAS ARCH Collaborative is a group of hospital organizations that are working together to measure and improve clinician EHR user experience. KLAS Research designed an EHR Experience Survey and works with individual hospitals or hospitals that are part of health systems to conduct it in a consistent manner.^13^ Each hospital site sent the survey to their full medical staff (ie, credentialed staff) of each organization or hospital. This survey included only clinician (ie, physician, nurse practitioner, and physician assistants) responses and was sent to their primary contact. Participating hospital organizations invited all their clinicians to participate through an emailed survey, with nonrespondents receiving a total of 3 follow-up emails. The mean response rate per organization was 18% (range, 2%-52%; IQR, 7%-24%). The standard survey questions can be found elsewhere.^13,14^

The core of the ARCH measurement survey was patterned on the System Usability Score questionnaire but was modified to be specific to the health care field.^13^ Like the System Usability Score questionnaire, respondents are asked on a 5-point scale from strongly agree (1) to strongly disagree (5) about the following 8 statements: (1) this EHR enables me to deliver high-quality care (quality care); (2) this EHR makes me as efficient as possible (efficiency); (3) this EHR is available when I need it (has almost no downtime) (reliability); (4) this EHR has the functionality for my specific specialty/clinical care area (functionality); (5) this EHR provides expected integration within our organization (internal integration); (6) this EHR provides expected integration with outside organizations (external integration); (7) this EHR has the fast system response time I expect (response time); and (8) this EHR is easy to learn (easy to learn).

### Study Sample

Approximately 1500 hospitals annually complete the Leapfrog EHR/CPOE Safety Evaluation Test, and 297 hospitals participate in the ARCH Collaborative. Our sample for this study included the 127 hospitals that took the Leapfrog Hospital Survey, including the EHR/CPOE Evaluation Tool, in at least 1 year from 2017 to 2018 and also participated in the KLAS ARCH Collaborative (ie, collected survey data) in the same years as the Leapfrog Hospital Survey EHR/CPOE Evaluation Test. Of these 127 hospitals, 15 were excluded from the final analysis because of incomplete ARCH Survey Data. Across these hospitals, we limited the ARCH survey sample to physicians only.

### Statistical Analysis

In this cross-sectional study, we first assessed the association of the hospital’s overall Leapfrog EHR/CPOE test score with the overall KLAS ARCH experience score at the individual respondent level. The Leapfrog overall and subcomponent scores were taken directly from the Leapfrog Hospital Survey results. Our KLAS ARCH experience score analytic data set was at the individual survey respondent level, with responses nested within hospitals. Hospital-level covariates included were region, size (number of beds), teaching status, for-profit status, and year of measurement; hospital characteristics were sourced from Leapfrog Hospital Survey data. The overall ARCH experience score was calculated by averaging the individual’s responses across all 8 questions. All ARCH subcomponents were equally weighted in the survey analysis.

We used an ordinary least squares regression with the Leapfrog score as the dependent variable and the KLAS ARCH score as the focal independent variable. SEs were clustered at the hospital level.

In the second model, we reestimated the model with the focal independent variables as the 8 ARCH subcategory experience scores to assess whether there were independent associations between each dimension of user experience and overall Leapfrog EHR/CPOE Test scores. Our third set of models then examined the associations of each Leapfrog subcategory score with the overall ARCH experience score, again using the aforementioned model specification.

To assess the robustness of our results to varied modeling approaches, we also ran specifications that (1) removed the control variables, (2) removed nesting by hospital, and (3) specified the ARCH experience scores as binary (ie, 1, agree or strongly agree; 0, indifferent, disagree, or strongly disagree). For further details see the eAppendix in Supplement 1. We used R statistical software version 3.2.2 extension PLM (R Project for Statistical Computing). Statistical significance was set at 2-sided *P* < .05. Data analysis was performed from September 2020 to November 2022.

## Results

A total of 112 hospitals and 5689 respondents are included in the sample. Table 1 reports overall hospital characteristics of the sample. The specialties of the respondents are shown in the eTable in Supplement 1. Hospitals from the western (34 hospitals [30%]) and southern (30 hospitals [27%]) US were most common, and nonprofit hospitals (101 hospitals [90%]) were the majority. Nonteaching hospitals were also the majority (88 hospitals [79%]), as were larger hospitals (>200 beds; 70 hospitals [62%]). Table 2 reports summary statistics for the overall Leapfrog EHR/CPOE score and the overall ARCH experience scores. The overall Leapfrog EHR/CPOE mean score was 0.673 (range, 0.297-0.973) on a scale of 0 to 1, with subcategory scores ranging from a low of 0.314 for drug-age to a high of 0.981 for drug-allergy. The overall ARCH experience score was 3.377 on a scale of 1 (best) to 5 (worst), with subcategory scores ranging from 2.778 for efficiency rating to 4.006 for reliability rating.

**Table 1.** Overall Hospital Descriptive Statistics

Characteristics	Hospitals, No. (%) (N = 112)
Region	
Midwest	17 (15)
Northeast	31 (28)
South	30 (27)
West	34 (30)
Ownership	
For profit	11 (10)
Nonprofit	101 (90)
Teaching	
Teaching	24 (21)
Nonteaching	88 (79)
Size, No. of beds	
1-100	16 (14)
101-200	26 (23)
201-400	36 (32)
≥401	34 (30)
Year of measurement	
2017	44 (39)
2018	68 (61)

**Table 2.** Hospital Data for Leapfrog and ARCH Scores

Scoring system and subdomain	Mean score
Leapfrog score (n = 112 hospitals)^a^	
Overall, mean (range)	0.673 (0.297-0.973)
Drug-route	0.940
Drug-allergy	0.981
Therapeutic duplication	0.704
Drug-dose daily	0.877
Drug-diagnosis	0.324
Drug-age	0.314
Drug-drug interaction	0.775
Drug-dose single	0.882
Drug-laboratory levels	0.524
Drug-monitoring	0.326
ARCH score (n = 5689 respondents)^b^	
Overall	3.377
Quality care	3.524
Efficiency	2.778
Reliability	4.006
Functionality	3.382
Response time	3.406
Easy to learn	3.148
External integration	3.070
Internal integration	3.698

^a^ The Leapfrog score is the number of questions on the test that the hospital got correct, expressed as a decimal from 0 to 1.

^b^ The ARCH score is a user experience rating ranging from 1 (best) to 5 (worst).

In the first model, we compared the overall leapfrog safety score with the overall ARCH experience score and found a β coefficient of 0.011 (95% CI, 0.006-0.016; *P* < .001) (Table 3). This means that a 1-point increase in the ARCH EHR Experience score (the difference between a clinician reporting that they agree vs strongly agree that the EHR was usable, efficient, integrated, and so forth) was associated with a 1.1 percentage point increase in overall Leapfrog Safety score (where 0.01 is more than 1/20th of the IQR of hospital safety measurements). These same results were found whether the analysis was done with or without covariates, or at the overall hospital health system level (where each hospital health system was treated as a single observation) or at the individual clinician response level.

**Table 3.** Association of Leapfrog Overall Safety Score With Overall ARCH Experience Score

Variable^a^	β (95% CI)	*P* value
ARCH electronic health record experience	0.011 (0.006 to 0.016)	<.001
Region		
Northeast	0.145 (0.140 to 0.150)	<.001
South	0.094 (0.08 to 0.100)	<.001
West	0.149 (0.140 to 0.160	<.001
Hospital beds	0.000 (0.000 to 0.000)	.07
Nonteaching hospital	−0.042 (−0.050 to −0.030)	<.001
For-profit hospital	0.002 (−0.010 to 0.010)	.60
Year 2018	0.034 (0.030 to 0.040)	<.001

^a^ Data are from 5689 respondents within 112 hospitals. Region Central, teaching hospital, not-for-profit status, and year 2017 are reference categories.

In the second model (Table 4), we compared the overall Leapfrog EHR/CPOE Score with the subcategory ARCH experience scores and found an association between all ARCH experience questions and the Leapfrog overall safety score, except for the reliability and quality questions. The ARCH survey questions with the largest coefficients were those measuring functionality (β, 0.007; 95% CI, 0.003 to 0.010), efficiency (β, 0.007; 95% CI, 0.004 to 0.011), and ease of learning (β, 0.007; 95% CI, 0.003 to 0.011). This could indicate that clinicians see safety in solutions that support strong adoption, functionality, and efficiency. Many comments from clinicians in the survey indicate a high level of frustration in a perceived tradeoff between efficiency and safety in the EHR, indicating that clinicians feel that inefficient and difficult to use systems are perceived to be more likely to induce stress and, thus, lead to mistakes.

**Table 4.** Models of the Association of Leapfrog Overall Score (Dependent Variable) With Individual Experience Questions (Primary Independent Variables)^a^

Primary covariate: ARCH electronic health record experience questions	β (95% CI)^b^	*P* value
Satisfaction quality care rating	0.003 (−0.001 to 0.007)	.02
Satisfaction external integration rating	0.006 (0.003 to 0.010)	<.001
Satisfaction functionality rating	0.007 (0.003 to 0.010)	<.001
Satisfaction efficiency rating	0.007 (0.004 to 0.011)	<.001
Satisfaction reliability rating	0.002 (−0.003 to 0.006)	.23
Satisfaction system response time rating	0.006 (0.002 to 0.009)	<.001
Satisfaction internal integration rating	0.004 (0.000 to 0.008)	<.001
Satisfaction easy to learn rating	0.007 (0.003 to 0.011)	<.001

^a^ Data are from 5689 respondents within 112 hospitals.

^b^ All 95% CIs are Bonferroni corrected.

In the third model (Table 5), we compared the overall ARCH experience score at the individual respondent level with the Leapfrog Safety Subcategory scores and found significant associations between the overall ARCH user experience score and all the Leapfrog subcategory scores except for drug-diagnosis. We found this association for every tested combination except 3 categories, with negative coefficients for the overall ARCH EHR experience score when regressed against the drug-laboratory, drug-diagnosis, and drug-monitoring Leapfrog subcategory variables. These Leapfrog subcomponents are the lowest performing subcategories in the Leapfrog EHR/CPOE test for both this study sample and all hospitals using the Leapfrog Test as well. In the associations of individual Leapfrog subcategory variables (Table 5), the largest coefficient was found for drug-drug interactions (β, 0.047; 95% CI, 0.031-0.062), followed by therapeutic duplication (β, 0.029; 95% CI, 0.014-0.045), drug-age (β, 0.021; 95% CI, 0.005-0.036), and drug-dose (β, 0.018; 95% CI, 0.006 to 0.031). This could indicate that clinicians especially value well-designed decision support catching these medication safety categories. To assess the robustness of these 3 models, we also ran specifications that (1) removed the control variables, (2) removed nesting by hospital health system, and (3) modeled the ARCH experience scores as binary instead of on a numerical scale (ie, 1, agree or strongly agree; and 0, indifferent, disagree, or strongly disagree) of EHR experience and we did not find any significant differences in the models.

**Table 5.** Models of the Association of the Component Leapfrog Scores (Dependent Variables) With the Overall KLAS Experience Average Score (Primary Independent Variable)

Dependent variable: Leapfrog electronic health record component score	β (95% CI)	*P* value
Drug-route	0.013 (0.006 to 0.020)	<.001
Drug-allergy	0.008 (0.002 to 0.014)	<.001
Therapeutic duplication	0.029 (0.014 to 0.045)	<.001
Drug-dose daily	0.018 (0.006 to 0.031)	<.001
Drug-diagnosis	−0.008 (−0.024 to 0.009)	.15
Drug-age	0.021 (0.005 to 0.036)	<.001
Drug-drug interaction	0.047 (0.031 to 0.062)	<.001
Drug-dose single	0.020 (0.009 to 0.032)	<.001
Drug-laboratory	−0.014 (−0.027 to −0.001)	.001
Drug-monitoring	−0.016 (−0.029 to −0.003)	<.001

^a^ Data are from 5689 respondents within 112 hospitals.

## Discussion

In this cross-sectional study, we found that frontline EHR user experiences of usability were associated with the hospital safety performance of their EHR. The Leapfrog EHR/CPOE saftey measurements primarily focus on a single critical part of the overall EHR use process—prescriber medication ordering. There was a significant association between the safety of the operational EHR and the experience that frontline clinicians have in using it, probably related in part to the frustration that they experience with medication ordering in poorly designed EHRs. For example, poorly designed EHR medication ordering may involve too many clicks and too many alerts that would not only frustrate physicians but also cause them to ignore the alerts.

There are few data evaluating frontline users’ perceptions of usability and the actual safety performance of EHRs.^3,4^ We found the largest association between user experience for the ARCH survey areas of external integration, functionality, efficiency, time, and ease of learning, which is not surprising because these areas reflect the users’ actual experience with the systems in terms of satisfaction, efficiency, and analytics ratings. The next highest association was with user internal integration and quality of care rating. The least associated was user reliability rating. Given the design and organization of the ARCH Collaborative user survey, these associations seem to reflect a logical model of increasing association between safety performance and frontline user satisfaction.

When we evaluated individual categories of the Leapfrog EHR/CPOE test with the user survey, we found that certain categories of safety were associated with the overall user satisfaction score, especially drug-drug interactions, drug-allergy, therapeutic duplication, drug-age, drug dosing, and drug-route; other categories were not associated, such as drug-diagnosis, drug-laboratory, or drug-monitoring. This may be because the former categories are those with higher overall scores in the safety test and the latter categories have had consistently lower scores.

We did not attempt to find direct causation between user satisfaction and safety performance of EHRs, nor would our data necessarily support a causative link. However, we speculate that this link may represent an association between how well a hospital implements and optimizes their EHR for both user satisfaction and safety performance. Several previous studies^15,16^ have suggested that hospitals that focus efforts on optimization of their EHRs beyond the basic implementation achieve greater value from their EHRs in terms of cost reduction, quality improvements, or safety enhancement. Prior work^7,8,9^ has also shown that the longer an EHR is implemented, the greater the user satisfaction. Without a doubt, one of the key improvements is in the usability of the system from a user perspective.^7,8,9^ As organizations continually optimize their EHR systems, often in response to users’ complaints about usability, it is important that they consider the usability changes in concert with the likely impact on safety of the system. They can use the Leapfrog EHR/CPOE tool as one method to assess the impact of their usability enhancements on the safety of the system. Indeed, our study would suggest that focusing on usability alone without also measuring concurrently the impact on the safety performance of the EHR system might compromise the safety of the overall system.

One challenge is that although both vendors and hospitals are struggling to improve the usability of their EHRs, they are often doing this in separate silos. This occurs, in part, because the federal agency responsible for EHRs’ oversight has developed a certification approach that applies to vendors only and not to implementers, such as hospitals, and users, such as front-line clinicians. The Office of the National Coordinator (ONC) for Health IT has developed testing standards for usability of EHRs as part of its vendor product certification program, but no part of this program applies to operational EHR software as implemented in health care organizations. Most of the off-the-shelf EHR vendor software is highly configured by local organizations before it is operationalized by users. Thus, the usability of software in operation may, and often does, differ greatly from the usability of the software on the shelf that was certified.^9,10,11,17^

This same challenge of software usability has also been faced by aviation, where airline manufacturers, airlines, and the Federal Aviation Administration work closely to continuously monitor and improve the software that increasingly controls airplanes. This shared responsibility led to great improvements in safety and pilot satisfaction with airplanes and their software. The importance of this shared responsibility has been highlighted by the recent Boeing software problems that brought down 2 airplanes.^18^ Much like the Boeing software issues, usability problems may be the initial indication of potential safety problems. EHR usability, which is the extent to which EHRs support clinicians in achieving their goals in a satisfying, effective, and efficient manner, is a point of continuing frustration for clinicians and can have patient safety consequences, as we and others have shown. EHR usability has almost certainly contributed to some serious patient harm events.^19,20,21^ Indeed, EHR usability complaints have led to safety investigation of 2 EHR vendors by the ONC and a resultant legal settlement for hundreds of millions of dollars by 2 vendors.^19^ EHR usability and safety improvements will require a more in-depth understanding of optimal processes to ensure safe and usable EHRs on the part of both the vendor and the user, which is not currently the case.^17,19,20^

In a landmark report on health information technology safety,^2^ the Institute of Medicine called for a similar type of shared responsibility as in aviation for use in health care software. One example of how such a shared responsibility between EHR vendors and frontline users could work is the new Life Cycle Health IT Safety Standards for EHR and Other Health IT Software^22^ recently released by Association for Advancement of Medical Instrumentation, a private sector standards development organization that previously developed the software safety standards for medical devices. Using a model that tightly links vendors and frontline users in a group of standards that address quality systems, risk management, and usability or human factors for clinical software, this model would allow for a self-improving system that uses safety software best practices from other industries to create safer and more usable software. It is a model that could be adopted by the Food and Drug Administration and ONC to address the critical ongoing issues in EHR safety and usability. It has the potential framework for enhanced oversight of EHR software by ONC, Food and Drug Administration, and Centers for Medicare & Medicaid Services.^2^

### Limitations

This study was limited by several factors, including a sample that may not be representative of all US hospitals with EHRs. The voluntary nature of both the Leapfrog Safety Survey and the ARCH collaborative may select for hospitals that are more likely to have better performance in both surveys, thus potentially lessening the association between these 2 hospital surveys. Future investigations should examine the association between user satisfaction and actual adverse drug events.

## Conclusions

We found a positive association between the safety performance of EHRs using an objective nationally endorsed standard and frontline users’ perceptions of EHR usability and experience. Both health systems and vendors need to consider usability not only as critical for the frontline users, but also as a critical safety issue in the design, development, implementation, and maintenance of these complex EHR systems, and they should work together with frontline users and organizations to improve usability without compromising the integrity of safety performance.

## Notes

Supplement 1. **eTable.** ARCH Survey Provider Respondent Specialty **eAppendix.** Further Details of Statistical Analysis

Supplement 2.
Data Sharing Statement

## References

[1] Classen DC, Holmgren AJ, Co Z, . National trends in the safety performance of electronic health record systems from 2009 to 2018. JAMA Netw Open. 2020;3(5):e205547. doi:10.1001/jamanetworkopen.2020.5547 32469412 PMC7260621

[2] Committee on Patient Safety and Health Information Technology; Institute of Medicine. Health IT and Patient Safety: Building Safer Systems for Better Care. National Academies Press; 2011. 24600741

[3] Howe JL, Adams KT, Hettinger AZ, Ratwani RM. Electronic health record usability issues and potential contribution to patient harm. JAMA. 2018;319(12):1276-1278. doi:10.1001/jama.2018.1171 29584833 PMC5885839

[4] Zahabi M, Kaber DB, Swangnetr M. Usability and safety in electronic medical records interface design: a review of recent literature and guideline formulation. Hum Factors. 2015;57(5):805-834. doi:10.1177/0018720815576827 25850118

[5] Phansalkar S, Zachariah M, Seidling HM, Mendes C, Volk L, Bates DW. Evaluation of medication alerts in electronic health records for compliance with human factors principles. J Am Med Inform Assoc. 2014;21(e2):e332-e340. doi:10.1136/amiajnl-2013-002279 24780721 PMC4173170

[6] Seidling HM, Phansalkar S, Seger DL, . Factors influencing alert acceptance: a novel approach for predicting the success of clinical decision support. J Am Med Inform Assoc. 2011;18(4):479-484. doi:10.1136/amiajnl-2010-000039 21571746 PMC3128393

[7] Shanafelt TD, Dyrbye LN, Sinsky C, . Relationship between clerical burden and characteristics of the electronic environment with physician burnout and professional satisfaction. Mayo Clin Proc. 2016;91(7):836-848. doi:10.1016/j.mayocp.2016.05.007 27313121

[8] Ellsworth MA, Dziadzko M, O’Horo JC, Farrell AM, Zhang J, Herasevich V. An appraisal of published usability evaluations of electronic health records via systematic review. J Am Med Inform Assoc. 2017;24(1):218-226. doi:10.1093/jamia/ocw046 27107451 PMC7654077

[9] Khajouei R, Farahani F. A combination of two methods for evaluating the usability of a hospital information system. BMC Med Inform Decis Mak. 2020;20(1):84. doi:10.1186/s12911-020-1083-6 32366248 PMC7199374

[10] Wolfstadt JI, Gurwitz JH, Field TS, . The effect of computerized physician order entry with clinical decision support on the rates of adverse drug events: a systematic review. J Gen Intern Med. 2008;23(4):451-458. doi:10.1007/s11606-008-0504-5 18373144 PMC2359507

[11] Ratwani RM, Savage E, Will A, . A usability and safety analysis of electronic health records: a multi-center study. J Am Med Inform Assoc. 2018;25(9):1197-1201. doi:10.1093/jamia/ocy088 29982549 PMC7646875

[12] Pruitt Z, Howe JL, Krevat SA, Khairat S, Ratwani RM. Development and pilot evaluation of an electronic health record usability and safety self-assessment tool. JAMIA Open. 2022;5(3):ooac070. doi:10.1093/jamiaopen/ooac070 35919379 PMC9338455

[13] Longhurst CA, Davis T, Maneker A, ; ARCH Collaborative. Local investment in training drives electronic health record user satisfaction. Appl Clin Inform. 2019;10(2):331-335. doi:10.1055/s-0039-1688753 31091545 PMC6520075

[14] KLAS Research. ARCH Collaborative. Accessed August 9, 2023. https://klasreseARCH.com/ARCH-collaborative

[15] Chaparro JD, Classen DC, Danforth M, Stockwell DC, Longhurst CA. National trends in safety performance of electronic health record systems in children’s hospitals. J Am Med Inform Assoc. 2017;24(2):268-274. doi:10.1093/jamia/ocw134 27638908 PMC7651940

[16] Metzger J, Welebob E, Bates DW, Lipsitz S, Classen DC. Mixed results in the safety performance of computerized physician order entry. Health Aff (Millwood). 2010;29(4):655-663. doi:10.1377/hlthaff.2010.0160 20368595

[17] Hettinger AZ, Melnick ER, Ratwani RM. Advancing electronic health record vendor usability maturity: progress and next steps. J Am Med Inform Assoc. 2021;28(5):1029-1031. doi:10.1093/jamia/ocaa329 33517394 PMC8068416

[18] Herkert J, Borenstein J, Miller K. The Boeing 737 MAX: lessons for engineering ethics. Sci Eng Ethics. 2020;26(6):2957-2974. doi:10.1007/s11948-020-00252-y 32651773 PMC7351545

[19] Ratwani RM, Benda NC, Hettinger AZ, Fairbanks RJ. Electronic health record vendor adherence to usability certification requirements and testing standards. JAMA. 2015;314(10):1070-1071. doi:10.1001/jama.2015.8372 26348757

[20] Ratwani RM, Savage E, Will A, . Identifying electronic health record usability and safety challenges in pediatric settings. Health Aff (Millwood). 2018;37(11):1752-1759. doi:10.1377/hlthaff.2018.0699 30395517

[21] Carspecken CW, Sharek PJ, Longhurst C, Pageler NM. A clinical case of electronic health record drug alert fatigue: consequences for patient outcome. Pediatrics. 2013;131(6):e1970-e1973. doi:10.1542/peds.2012-3252 23713099

[22] Association for the Advancement of Medical Instrumentation. American national standard: safety and effectiveness of health IT software and systems. Part 4: Application of human factors engineering. July 7, 2020. Accessed August 9, 2023. https://array.aami.org/doi/abs/10.2345/9781570207594.ch1

