# Supplementary material for: Inpatient EHR User Experience and Hospital EHR Safety Performance
Source: JAMA Netw Open. 2023 Sep 11;6(9):e2333152. doi: 10.1001/jamanetworkopen.2023.33152 (PMC10495862; doi:10.1001/jamanetworkopen.2023.33152)
Supplement: Supplement 1. — eTable. ARCH Survey Provider Respondent Specialty eAppendix. Further Details of Statistical Analysis [file jamanetwopen-e2333152-s001.pdf]

Supplemental Online Content

Classen D, Longhurst CA, Davis T, Milstein JA, Bates DW. Inpatient EHR user experience and hospital EHR safety performance. *JAMA Netw Open*. 2023;6(9):e2333152. doi:10.1001/jamanetworkopen.2023.33152

eTable. ARCH Survey Provider Respondent Specialty
eAppendix. Further Details of Statistical Analysis

This supplemental material has been provided by the authors to give readers additional information about their work.

© 2023 Classen D et al. *JAMA Network Open.*

eTable. ARCH Survey Provider Respondent Specialty

Specialty	N	%
Anesthesiology	231	4%
Cardiology	233	4%
Cardiothoracic surgery	33	1%
Critical care	60	1%
Dermatology	42	1%
Emergency medicine	485	9%
Endocrinology	70	1%
Family medicine	383	7%
Gastroenterology	79	1%
General surgery	234	4%
Geriatrics	47	1%
Gynecology and obstetrics	316	6%
Hematology/oncology	95	2%
Hospital medicine	387	7%
Infectious disease	116	2%
Internal medicine	446	8%
Multiple Specialties	357	6%
Neonatology	52	1%
Nephrology	71	1%
Neurology	143	3%
Neurosurgery	44	1%
Oncology	48	1%
Ophthalmology	46	1%
Orthopedics	179	3%
Other (specialty not on this list)	295	5%
Otolaryngology	53	1%
Pathology	62	1%
Pediatrics	387	7%
Physical rehabilitation	36	1%
Plastic surgery	51	1%
Psychiatry	101	2%
Pulmonology	70	1%
Radiology	196	3%
Rheumatology	33	1%
Urology	57	1%
Vascular surgery	23	0%
Unknown Specialty	128	2%

© 2023 Classen D et al. *JAMA Network Open.*

eAppendix. Further Details of Statistical Analysis

With respect to Table 5 we also completed the same regression with pooled errors by hospital but excluded all other covariates. Doing this, our results were very similar to our initial findings with the included covariates. The covariate for the regression of the overall Arch Experience Score and the Leapfrog Overall Safety score is nearly identical as that of Table 3 (beta= .012 vs. .011 in table 3), with both being statistically significant with p<.001.

We also completed the same series of regressions as in Table 5 with the Arch Collaborative variables structured as binary variables, with an answer of Strongly Agree/Agree categorized as ‘1’ and other answers (Neutral/Disagree/Strongly Disagree) categorized as ‘0’. The results from this series of regressions were also nearly identical to the primary regression in Table 5, with different coefficients reflecting the change in model specification, but every regression found to be statistically significant (p<.05).

We also completed a series of simple regressions with the hospital Leapfrog safety scores regressed against the average hospital Arch Collaborative overall score, similar to Tables 3 and 5. This model ignored the power of the multiple responses per hospital, and would be expected to have lower significance, which is exactly what we found. However, with only 112 observations, and including other covariates, the correlation between the Overall Leapfrog Safety Score and the Overall Arch Collaborative score was statistically significant (p<.01) with actually a larger coefficient (B = .1).

© 2023 Classen D et al. *JAMA Network Open.*
