# Supplementary material for: Inpatient EHR User Experience and Hospital EHR Safety Performance
Source: JAMA Netw Open. 2023 Sep 11;6(9):e2333152. doi: 10.1001/jamanetworkopen.2023.33152 (PMC10495862; doi:10.1001/jamanetworkopen.2023.33152)
Supplement: Supplement 2. — Data Sharing Statement [file jamanetwopen-e2333152-s002.pdf]

Data Sharing Statement

Classen. Inpatient EHR User Experience and Hospital EHR Safety Performance. *JAMA Netw Open*. Published September 11, 2023. doi:10.1001/jamanetworkopen.2023.33152

Data
Data available: No

Additional Information
Explanation for why data not available: there is no patient data in this study and the only data is taken from surveys and simulation tests which have not been formatted for public access but if the editors wish we could discuss ways to make the data available
